# Gut Microbiota and Liver Fibrosis: One Potential Biomarker for Predicting Liver Fibrosis

**DOI:** 10.1155/2020/3905130

**Published:** 2020-06-19

**Authors:** Zhiming Li, Ming Ni, Haiyang Yu, Lili Wang, Xiaoming Zhou, Tao Chen, Guangzhen Liu, Yuanxiang Gao

**Affiliations:** ^1^Department of Radiology, The Affiliated Hospital of Qingdao University, Qingdao, China 266000; ^2^Department of Pathology, The Affiliated Hospital of Qingdao University, Qingdao, China 266000

## Abstract

**Purpose:**

To investigate the relationship between gut microbiota and liver fibrosis and establish a microbiota biomarker for detecting and staging liver fibrosis.

**Methods:**

131 Wistar rats were used in our study, and liver fibrosis was induced by carbon tetrachloride. Stool samples were collected within 72 hours after the last administration. The V4 regions of 16S rRNA gene were amplified. The sequencing data was processed using the Quantitative Insights Into Microbial Ecology (QIIME version 1.9). The diversity, principal coordinate analysis (PCoA), nonmetric multidimensional scaling (NMDS), and linear discriminant analysis (LDA) effect size (LEfSe) were performed. Random-Forest classification was performed for discriminating the samples from different groups. Microbial function was assessed using the PICRUST.

**Results:**

The Simpson in the control group was lower than that in the liver fibrosis group (*p* = 0.048) and differed significantly among different fibrosis stages (*p* = 0.047). The Chao1 index in the control group was higher than that in the liver fibrosis group (*p* < 0.001). NMDS analysis showed a marked difference between the control and liver fibrosis groups (*p* < 0.001). PCoA analysis indicated the different community composition between the control and liver fibrosis groups with variances of PC1 13.76% and PC2 5.89% and between different liver fibrosis stages with variances of PC1 10.51% and PC2 7.78%. LEfSe analysis showed alteration of gut microbiota in the liver fibrosis group. Biomarkers obtained from Random-Forest classification showed excellent diagnostic accuracy in prediction of liver fibrosis with AUROCs of 0.99. The AUROCs were 0.77~0.84 in prediction of stage F4. There were six increased and 17 decreased metabolic functions in the liver fibrosis group and 6 metabolic functions significantly differed among four liver fibrosis stages.

**Conclusion:**

Gut microbiota is a potential biomarker for detecting and staging liver fibrosis with high diagnostic accuracies.

## 1. Introduction

The crosstalk between gut and liver is deduced by the gut-liver axis [1]. They are connected by bile acids and portal venous system. A number of liver diseases including nonalcoholic fatty liver disease (NAFLD), liver cirrhosis and cancer have a strong link with gut microbiota [2]. Chronic liver diseases (CLD) can lead to impaired gastrointestinal motility, spontaneous bacterial peritonitis, portal hypertension, and intestinal bacteria overgrowth. Recently, one gut microbiome analysis study validated that the composition of gut bacterial species altered in patients with liver cirrhosis [3]. A high prevalence of 40.8% for small intestinal bacterial overgrowth was found in patients with CLD [4] and even 50.5% for patients with decompensated cirrhosis. However, these studies focused on advanced liver disease, i.e., liver cirrhosis.

Liver fibrosis is characterized by excessive deposition of extracellular matrix (ECM). The activation of hepatic stellate cells (HSCs) is crucial for liver fibrogenesis. The dysfunction of the intestinal barrier in CLD patients resulted in the increased translocation of intestinal bacteria and their components [5]. Due to continuous exposure of lipopolysaccharide (LPS) from gram-negative bacteria of the gut microbiota, transforming growth factor- (TGF-) *β* signaling in HSCs was activated by Toll-like receptor (TLR) 4-mediated innate immunity [6]. Therefore, gut microbita in CLD patients promotes liver fibrosis. Because liver fibrogenesis is an ongoing process, changes of gut microbiota associated with liver fibrosis stages are still unrevealed in detail.

To investigate the relationship between gut microbiota and liver fibrosis, we used 16S rRNA Amplicon Pyrosequencing to disclose the alteration of microbiota composition in liver fibrosis rats. The purpose of the study was to establish a microbiota biomarker for detecting and staging liver fibrosis.

## 2. Materials and Methods

### 2.1. Induced Liver Fibrosis Rat Model

131 specific pathogen-free Wistar rats (male, 200 ± 50 g) were used in our study and were housed in our institutional animal laboratory with a room temperature of 25°C and room humidity of 50%. Rats of the liver fibrosis group were subcutaneously administrated twice a week with 0.3 ml/100 g mixed solution, which was composed of carbon tetrachloride (CCl_4_) and olive oil at a ratio of 1 : 1. Rats of the control group were treated with saline.

### 2.2. Stool Sample Collection and DNA Extraction

All stool samples of both liver fibrosis and control groups were collected within 72 hours after the last CCl_4_ or saline administration and stored in a stool container with a novel chemical stabilizer, i.e., N-octylpyridinium bromide- (NOPB-) based reagent. It allows stool sample transportation and storage at room temperature with preservation of bacterial composition, and the composition and performance of NOPB-based reagent were described in detail by Han et al. [7]. Total DNA was extracted from stool samples by using the PowerMax (stool/soil) DNA isolation kit (MoBio Laboratories, Carlsbad, CA, United States). Then, DNA samples were stored at -20°C for further analysis. Extracted DNA concentrations were assessed with absorption ratios of DNA at 260/280 nm and 260/230 nm by using a NanoDrop 2000c spectrophotometer (Thermo Scientific, USA).

### 2.3. 16S rRNA Gene Sequencing

The V4 regions of 16S rRNA gene were amplified with the forward primer 515F (5′–GTGCCAGCMGCCGCGGTAA–3′) and the reverse primer 806R (5′–GGACTACHVGGGTWTCTAAT–3′). PCR amplification was performed in a mixture containing 25 *μ*l of Phusion High-Fidelity PCR Master Mix, 3 *μ*l of DMSO, 3 *μ*l of each forward and reverse primer, 10 *μ*l of DNA Template, and 6 *μ*l of ddH_2_O. Thermal cycling reaction was as the following: initial denaturation at 98°C for 30 s, followed by 30 cycles of denaturation at 98°C for 15 s, annealing at 58°C for 15 s, and extension at 72°C for 15 s, and final extension at 72°C for 1 min. PCR products were purified with AMPure XP Beads (Beckman Coulter, Indianapolis, IN). The amplicons were normalized, pooled, and sequenced on the Illlumina HiSeq 4000 sequencer (2 × 150 bp; Guhe Information and Technology Co., Ltd., Hangzhou, China).

### 2.4. Sequence Analysis and Statistical Analysis

The sequencing data was processed using the Quantitative Insights Into Microbial Ecology (QIIME version 1.9) [8]. The low-quality sequence reads were removed following the criteria: (1) reads with a length of <150 bp, (2) reads with an average Phred score of <20, (3) reads containing ambiguous bases, and (4) reads containing mononucleotide repeats of >8 bp. High-quality reads were clustered into 16S rRNA Operational Taxonomic Units (OTUs) with ≥97% sequence homology. The taxonomic classification of each OTU was performed by VSEARCH searching the representative sequence set against the SILVA reference database [9].

The R package (3.2.0) was applied to analyze the distribution of sequence length in all samples. OTU tables were used to record the abundance of each OTU of samples. Taxon abundance at the levels of phylum, class, order, family, genus, and species was calculated and statistically compared among groups using R stats package. Based on the OTU table in QIIME, alpha diversities including Chao1, Simpson, and Shannon were calculated. The significant differences of alpha diversity metrics were performed using the R package “Vegan.” The Venn diagram generated by R package was used to visualize the shared and unique OTUs among groups. In order to investigate the structural variation of microbial communities, beta diversity analysis was performed using UniFrac distance metrics [10, 11] and was visualized via principal component analysis (PCA), principal coordinate analysis (PCoA), and nonmetric multidimensional scaling (NMDS) [12]. The linear discriminant analysis (LDA) effect size (LEfSe) method based on the Kruskal-Wallis test and linear discriminant analysis (LDA) was conducted to identify significant differentially abundant taxonomy between different groups [13]. Random-Forest classification was performed for discriminating the samples from different groups using the R package “randomForest” [14, 15], and “pROC” package was used for receiver operating curve (ROC) analysis. Based on 16S rRNA marker gene sequences, microbial function was predicted using the PICRUST (phylogenetic investigation of communities by reconstruction of unobserved states) [16]. By means of the KEGG database, Welch's *t*-test was used to confirm the significant difference of function between the control and liver fibrosis groups with a *p* value less than 0.05. Differences of function between liver fibrosis stages were analyzed using the Kruskal test from R stats package.

## 3. Results

### 3.1. Liver Fibrosis Stages

Wistar rats were induced via injection of CCl_4_ for 4, 6, 8, and 12 weeks. Rats were sacrificed at 72 h after the last injection of CCl_4_. Liver species of rats were fixed in formalin and underwent hematoxylin-eosin staining and Masson's trichrome staining. According to the histopathologic analysis, liver fibrosis was categorized into five stages [17]: F0: no fibrosis; F1: fibrous portal expansion; F2: few bridges or septa; F3: numerous bridges or septa; and F4: cirrhosis.

**Figure 1:**
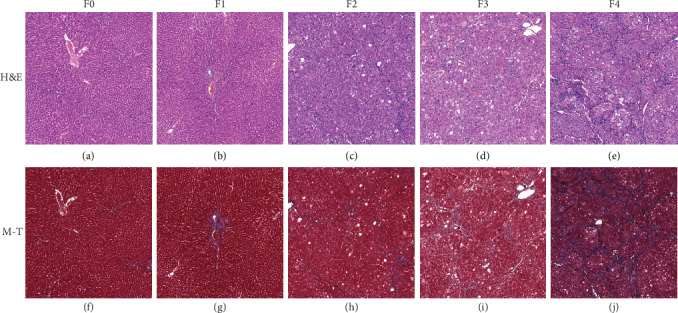
H&E and Masson's trichrome staining in liver fibrosis. Liver fibrosis stages: F0: no fibrosis (a, f); F1: fibrous portal expansion (b, g); F2: few bridges or septa (c, h); F3: numerous bridges or septa (d, i); F4: cirrhosis (e, j). M-T: Masson trichrome staining.

Finally, a total of 131 rats were enrolled in our study and were divided into the control group (*n* = 65) and liver fibrosis group (*n* = 66). As for the liver fibrosis group, there were 15 F1 rats, 22 F2 rats, 11 F3 rats, and 18 F4 rats (Figure 1).

### 3.2. Differences between Control and Liver Fibrosis Groups

**Figure 2:**
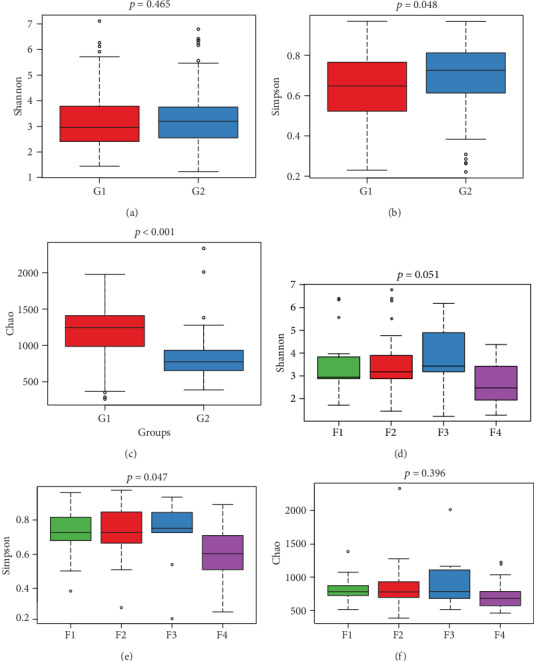
Differences of alpha diversity between the control and liver fibrosis groups and between different liver fibrosis stages. Between the control and liver fibrosis group, no difference of Shannon was found (*p* = 0.465) (a). The Simpson in the control group was lower than that in the liver fibrosis group (*p* = 0.048) (b). The Chao1 of the alpha diversity index in the control group was higher than that in the liver fibrosis group (*p* < 0.001) (c). Between different liver fibrosis stages, there were no differences of Shannon and Chao1 indices (d, f). Significant difference of Simpson was found with *p* = 0.047 (e). G1: control group; G2: liver fibrosis group; F1~F4: liver fibrosis stages.

No difference of Shannon was found between the control and liver fibrosis groups (*p* = 0.465) (Figure 2(a)). The Simpson in the control group was lower than that in the liver fibrosis group (*p* = 0.048) (Figure 2(b)). The Chao1 of the alpha diversity index in the control group was higher than that in the liver fibrosis group (*p* < 0.001) (Figure 2(c)).

**Figure 3:**
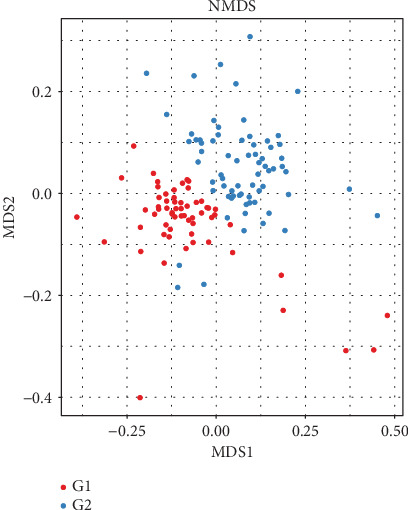
Nonmetric multidimensional scaling (NMDS) analysis between control and liver fibrosis. There was a marked difference between the control group and the liver fibrosis group (*p* < 0.001). G1: control group; G2: liver fibrosis group.

**Figure 4:**
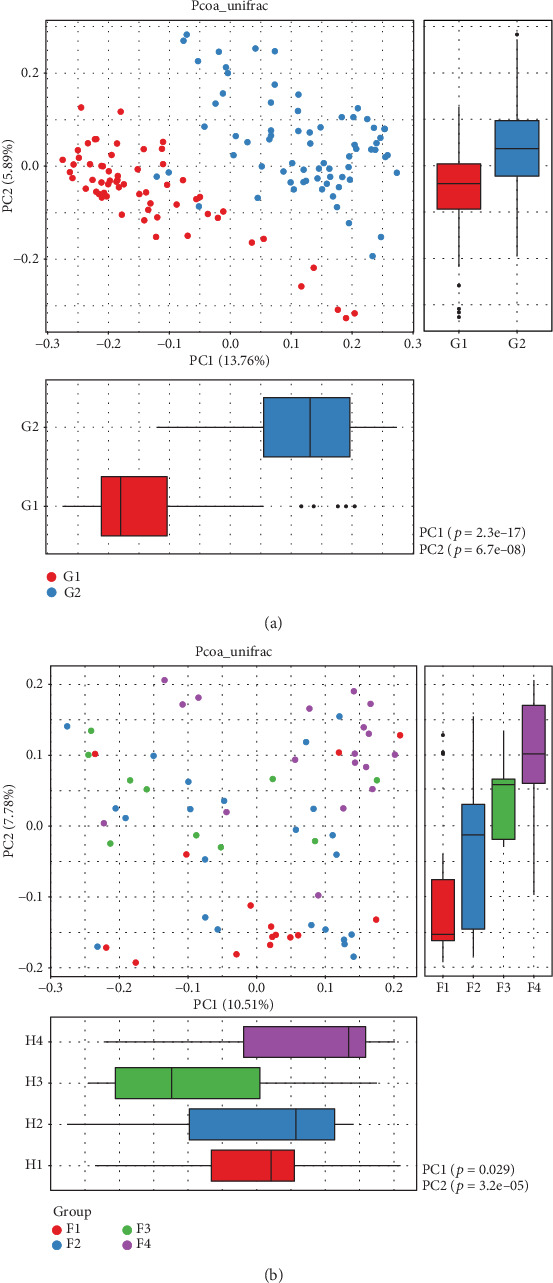
Principal coordinate analysis (PCoA) between the control and liver fibrosis groups and between different liver fibrosis stages. PCoA analysis indicated the different community composition in OUT-level between the control group and the liver fibrosis group with variances of PC1 13.76% and PC2 5.89% (a) and between four different fibrosis stages with variances of PC1 10.51% and PC2 7.78% (b). G1: control group; G2: liver fibrosis group.

NMDS analysis showed that there was a marked difference between the control group and the liver fibrosis group (*p* < 0.001) (Figure 3). By using unweighted UniFrac distance, PCoA analysis indicated the different community composition in OUT-level between the control group and the liver fibrosis group with variances of PC1 13.76% and PC2 5.89% (Figure 4(a)).

**Figure 5:**
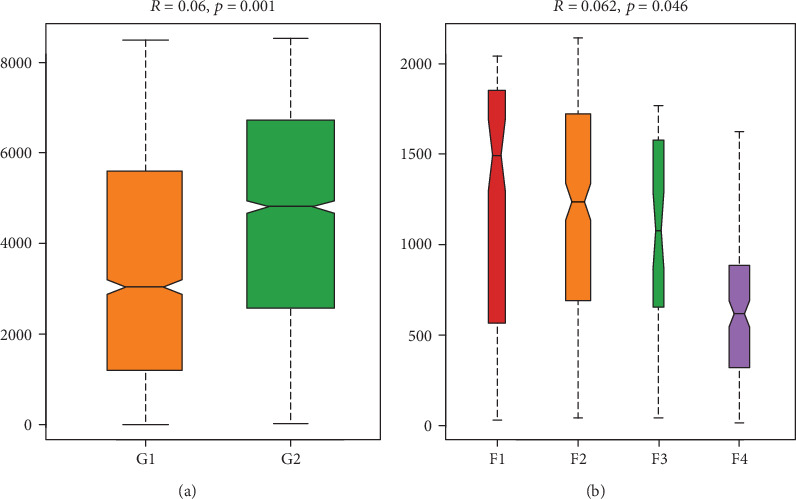
Analysis of similarities (ANOSIM) between the control and liver fibrosis groups and between different liver fibrosis stages. Significant difference of the bacteria community composition was found between the control and liver fibrosis groups (*p* = 0.001) (a) and between different liver fibrosis stages (*p* = 0.046) (b). G1: control group; G2: liver fibrosis group; F1~F4: liver fibrosis stages.

**Figure 6:**
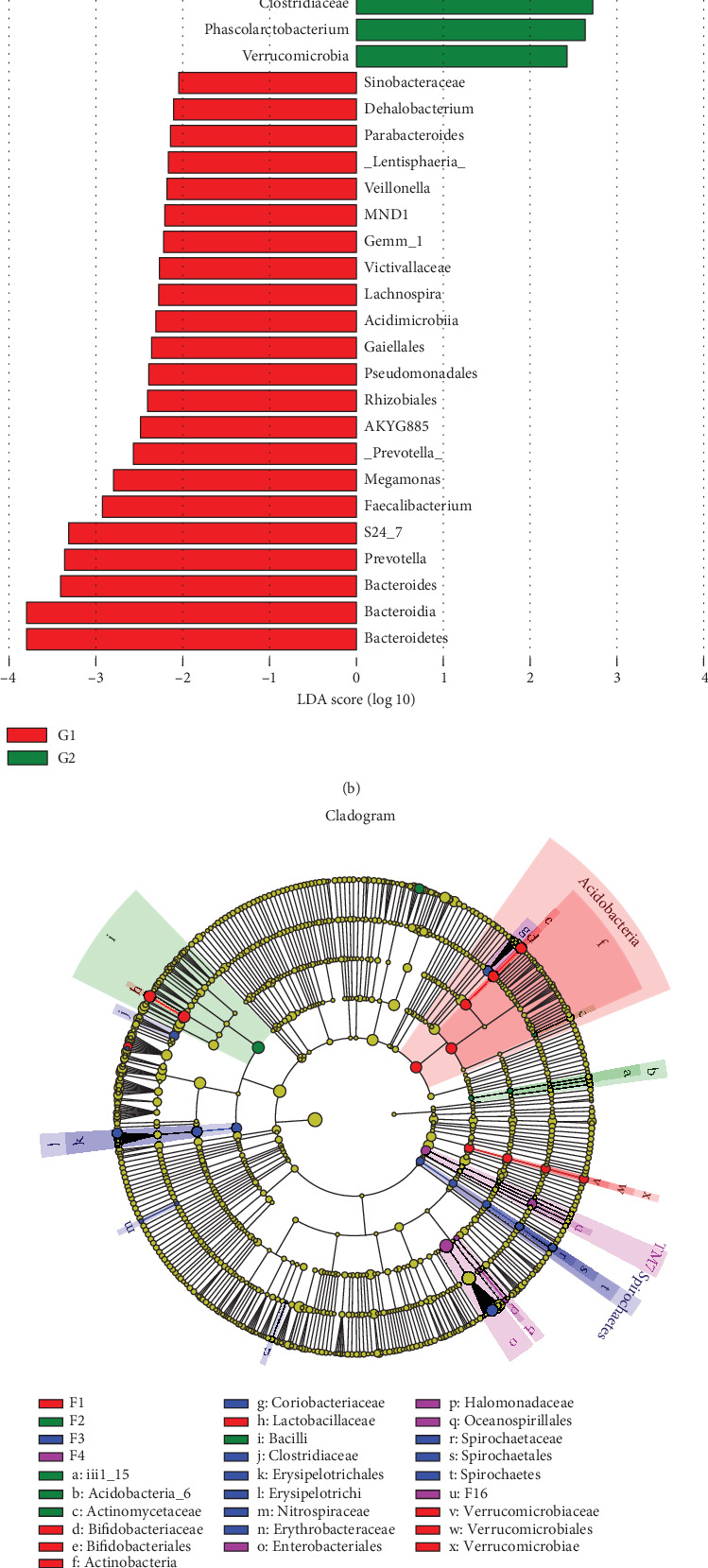
The linear discriminant analysis (LDA) effect size (LEfSe) analysis between the control and liver fibrosis groups and between different liver fibrosis stages. Cladogram (a, c) and the distinct bacterial species between the control and liver fibrosis groups and between different liver fibrosis stages, with a LAD score > 2 (b, d). G1: control group; G2: liver fibrosis group.

According to analysis of similarities (ANOSIM), a significant difference of the bacteria community composition was found between the control and liver fibrosis groups (*p* = 0.001) (Figure 5(a)). The LEfSe analysis was performed to identify the distinct bacterial species between the control and liver fibrosis groups with a LAD score > 2 (Figures 6(a) and 6(b)). At the phylum level, significantly increased relative abundance of *Actinobacteria*, *TM7*, and *Tenericutes* and decreased relative abundance of *Bacteroidetes* and *Verrucomicrobia* were found in the liver fibrosis group (*p* < 0.05). At the class level, higher abundance of *Actinobacteria*, *Coriobacteriia*, *Erysipelotrichi*, *TM7-3*, and *Mollicutes* and lower abundance of *Bacteroidia*, *Bacilli*, *Betaproteobacteria*, and *Verrucomicrobiae* were found in the liver fibrosis group (*p* < 0.05). At the order level, 6 orders of *Bifidobacteriales*, *Coriobacteriales*, *Turicibacterales*, *Erysipelotrichales*, *CW040*, and *RF39* increased significantly in the liver fibrosis group, and 4 orders of *Bacteroidales*, *Lactobacillales*, *Burkholderiales*, and *Verrucomicrobiales* decreased significantly (*p* < 0.05). At the family level, higher abundance of *Bifidobacteriaceae*, *Coriobacteriaceae*, *S24-7*, *Turicibacteraceae*, *Clostridiaceae*, *Lachnospiraceae*, *Erysipelotrichaceae*, and *F16* was identified in the liver fibrosis group and lower abundance of *Bacteroidaceae*, *Prevotellaceae*, *Paraprevotellaceae*, *Veillonellaceae*, *Alcaligenaceae*, and *Verrucomicrobiaceae* in the liver fibrosis group (*p* < 0.05). At the genus level, 9 genera including *Bifidobacterium*, *Adlercreutzia*, *CF231*, *Turicibacter*, *Clostridium*, *Dorea*, *Ruminococcus*, *Phascolarctobacterium*, and *Allobaculum* increased significantly in the liver fibrosis group, and 6 genera including *Bacteroides*, *Prevotella* (*Prevotellaceae*), *Prevotella* (*Paraprevotellaceae*), *Faecalibacterium*, *Megamonas*, and *Akkermansia* decreased significantly (*p* < 0.05).

**Figure 7:**
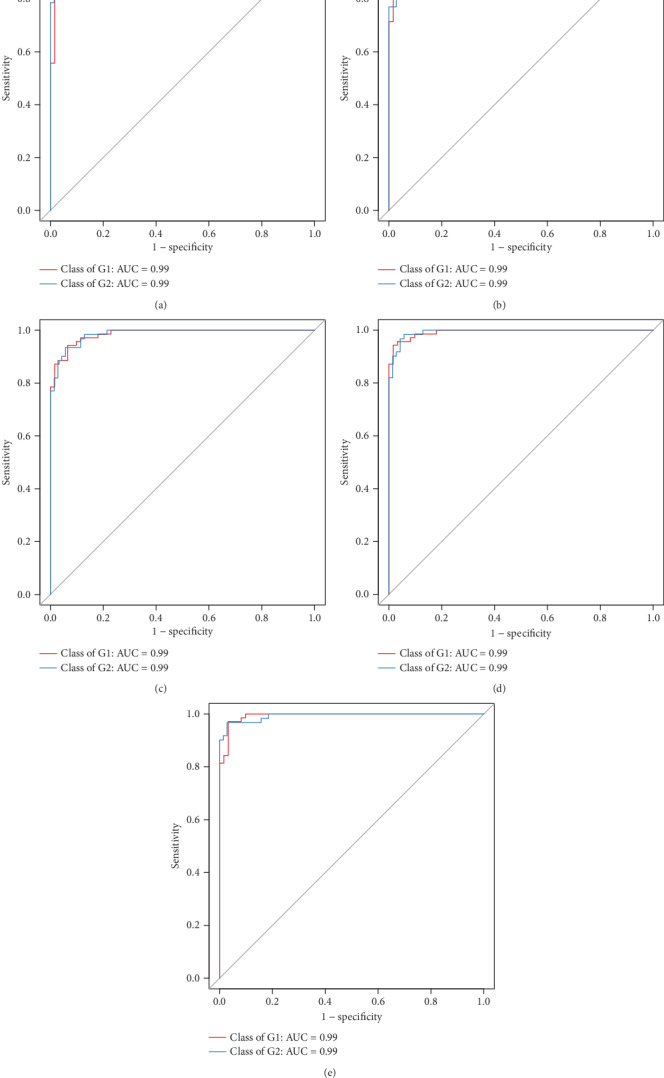
ROC analysis between the control group (G1) and the liver fibrosis group (G2). The areas under ROC at the phylum (a), class (b), order (c), family (d), and genus (e) were 0.99 for all in differentiating the control and liver fibrosis groups.

According to Random-Forest classification analysis, the top 60 species were selected, and the areas under ROC (AUROC) at the phylum, class, order, family, and genus were 0.99 for all in differentiating the control and liver fibrosis groups (Figure 7).

The PICRUST analysis results indicated that 23 KEGG pathways in level 3 were significantly different between the control and liver fibrosis groups. There were six increased metabolic functions in the liver fibrosis group including electron transfer carriers (unclassified), basal transcription factors, ether lipid metabolism, nucleotide metabolism, biosynthesis of stilbenoid diarylheptanoid and gingerol, and various types of N-glycan biosynthesis (*p* < 0.05). 17 decreased metabolic functions were identified in the liver fibrosis group including endocytosis, lysosome, cellular antigens, Notch signaling pathway, Wnt signaling pathway, mRNA surveillance pathway, 1,1,1-trichloro-2,2-bis(4-chlorophenyl)-ethane (DDT) degradation, biosynthesis of type II polyketide products, carotenoid biosynthesis, cytochrome P450, glycosaminoglycan degradation, glycosphingolipid biosynthesis (ganglio series), glycosphingolipid biosynthesis (globo series), glycosphingolipid biosynthesis (lacto and neolacto series), N-glycan biosynthesis, other glycan degradation, and steroid hormone biosynthesis (*p* < 0.05).

### 3.3. Differences among Different Liver Fibrosis Stages

No significant differences of Chao1 and Shannon were found among different fibrosis stage rats (*p* > 0.05) (Figures 2(d) and 2(f)). The Simpson of the alpha diversity index differed significantly among four different fibrosis stages (*p* = 0.047) (Figure 2(e)). PCoA analysis using unweighted UniFrac distance showed the different community composition in OUT-level among four different fibrosis stages with variances of PC1 10.51% and PC2 7.78% (Figure 4(b)).

According to ANOSIM, a significant difference in the bacteria community composition was found among different fibrosis stages (*p* = 0.046) (Figure 5(b)). To identify the distinct bacterial species among different fibrosis stages, the LEfSe analysis results were shown in Figures 6(c) and 6(d). At the phylum level, 3 phyla including *Actinobacteria*, *Proteobacteria*, and *TM7* differed significantly among four different fibrosis stages (*p* < 0.05). At the class level, 5 classes of *Actinobacteria*, *Bacilli*, *Erysipelotrichi*, *TM7-3*, and *Verrucomicrobiae* were distinct among four fibrosis stages (*p* < 0.05). At the order level, 5 orders of *Bifidobacteriales*, *Lactobacillales*, *Erysipelotrichales*, *CW040*, and *Verrucomicrobiales* differed significantly among four fibrosis stages (*p* < 0.05). At the family level, significant differences of *Bifidobacteriaceae*, *Lactobacillaceae*, *Veillonellaceae*, *Erysipelotrichaceae*, *F16*, and *Verrucomicrobiaceae* were found among four different fibrosis stages (*p* < 0.05). At the genus level, 7 genera of *Bifidobacterium*, *CF231*, *Lactobacillus*, *Dorea*, *Allobaculum*, *Morganella*, and *Akkermansia* were different significantly among four fibrosis stages (*p* < 0.05).

**Figure 8:**
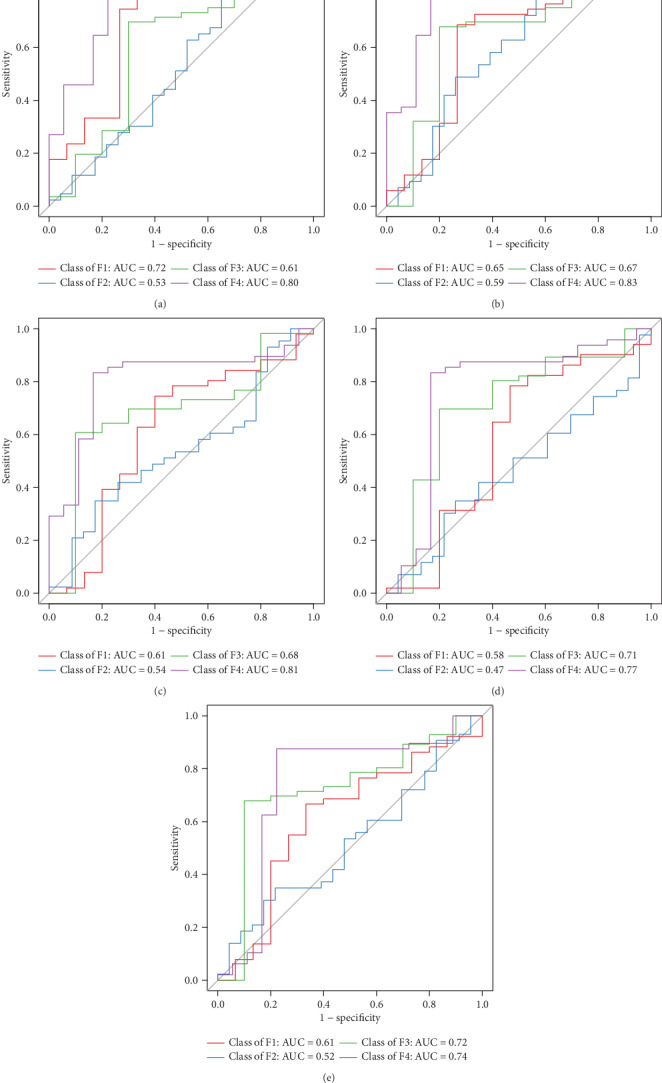
ROC analysis in prediction of stages F1, F2, F3, and F4. AUROCs in prediction of stages F1, F2, F3, and F4 were as follows: phylum level (a) 0.72, 0.53, 0.61, and 0.80; class level (b) 0.65, 0.59, 0.67, and 0.83; order level (c) 0.61, 0.54, 0.68, and 0.81; family level (d) 0.58, 0.47, 0.71, and 0.77; genus level (e) 0.61, 0.52, 0.72, and 0.84.

AUROCs in prediction of stages F1, F2, F3, and F4 at the phylum level were 0.72, 0.53, 0.61, and 0.80, respectively. At the class level, the AUROCs were 0.65, 0.59, 0.67, and 0.83. At the order level, the AUROCs were 0.61, 0.54, 0.68, and 0.81. At the family level, the AUROCs were 0.58, 0.47, 0.71, and 0.77. At the genus level, the AUROCs were 0.61, 0.52, 0.72, and 0.84 (Figure 8).

According to the PICRUST analysis, six metabolic functions of apoptosis, 1,1,1-trichloro-2,2-bis(4-chlorophenyl)ethane (DDT) degradation, betalain biosynthesis, ether lipid metabolism, fatty acid elongation in mitochondria, and indole alkaloid biosynthesis, were different significantly among different liver fibrosis stages (*p* < 0.05).

## 4. Discussion

In our study, the marked changes of gut microbiota were identified between normal liver rats and liver fibrosis rats. Furthermore, changes in gut microbiota were also found in four different liver fibrosis stages. On the basis of our results, it can help us to further understand the gut-liver axis.

Compared with normal liver rats, lower bacteria community richness (Chao1 index) and lower community diversity (Simpson index) were found in liver fibrosis rats in our study. A significant difference in bacteria community diversity (Simpson index) was found among four different fibrosis stages (*p* = 0.047). Moreover, the community diversity in stage F4 rats was higher than that in stages F1, F2, and F3, respectively. A number of liver diseases resulting in liver fibrosis can affect the richness and diversity of gut microbiota. As for the alcoholic liver disease (ALD), lower fungal species richness and diversity were confirmed in ALD patients compared with healthy controls [18]. Reduction in microbial diversity was found in patients with primary sclerosing cholangitis (PSC) [19, 20] and primary biliary cholangitis (PBC) [21]. HCV and HBV infection patients showed lower bacterial diversity [22, 23]. In addition, there is a decrease in microbial diversity in nonobese patients with NAFLD [24], and in patients with obesity and diabetes [25]. These studies mentioned above demonstrate the alteration of gut microbiota in different etiologies of liver fibrosis. However, these studies have not explained the correlation between gut microbiota and liver fibrosis. Recently, one study using a blood sample showed the lower bacterial 16S rDNA diversity in obese patients with liver fibrosis [26]. Undoubtedly, there is a significant difference between blood microbiomes and gut microbiomes [27]. Herein, our study fills this gap with encouraging results.

Remarkable biomarkers between normal liver rats and liver fibrosis rats were identified by using the LEfSe analysis in our study. There were three increased phyla and two decreased phyla, five increased and four decreased classes, six increased and four decreased orders, eight increased and six decreased families, and nine increased and six decreased genera. Moreover, we used the Random-Forest Classifier with AUROCs to predict the control group and the liver fibrosis group. At the phylum, class, order, family, and genus levels, biomarkers obtained from Random-Forest classification showed excellent diagnostic accuracies in differentiating between the control and liver fibrosis groups with AUROCs of 0.99 for all. Similarly, one recent study showed that using the Random-Forest Classifier model could distinguish mild/moderate from advanced liver fibrosis in NAFLD patients with an AUROC of 0.936 [28]. Furthermore, we also conducted Random-Forest classification to differentiate among liver fibrosis stages. Our results indicated that there was low diagnostic accuracy in the prediction of F2 with AUROC of less than 0.60. We speculated that there were overlaps between stages F1 and F2, and F2 and F3, in bacterial community diversity and composition. As to the prediction of stage F4, there were relatively high diagnostic accuracies with AUROC of 0.77~0.84 in our study.

The alterations of metabolic functions in gut microbiota were predicted by PICRUST analysis in our study. We found 6 increased and 17 decreased metabolic functions in the liver fibrosis group and 6 metabolic functions significantly differed among stages F1, F2, F3, and F4.

Our study disclosed the relationship between liver fibrosis and gut microbiota and successfully identified the biomarkers in predicting liver fibrosis. But there are some limitations including the relatively small sample size and a rodent animal study.

In conclusion, our study verifies that gut microbiota is a potential biomarker for detecting and staging liver fibrosis with high diagnostic accuracies.

## Data Availability

The data used to support the findings of this study are available from the corresponding author upon request.
